# Efficacy and safety of IL-23 inhibitors in the treatment of moderate to severe ulcerative colitis: a meta-analysis based on randomized controlled trials

**DOI:** 10.3389/fmed.2025.1724448

**Published:** 2025-12-04

**Authors:** Minyang He, Huixian Liang, Xiangcheng Fan, Leimin Sun

**Affiliations:** 1 Department of Gastroenterology, the Fourth Affiliated Hospital of School of Medicine, and International School of Medicine, International Institutes of Medicine, Zhejiang University, Yiwu, China; 2 Tongde Hospital of Zhejiang Province Affiliated to Zhejiang Chinese Medical University (Tongde Hospital of Zhejiang Province), Hangzhou, Zhejiang, China; 3 Zhejiang Academy of Traditional Chinese Medicine, Hangzhou, Zhejiang, China; 4 Department of Gastroenterology, Sir Run Run Shaw Hospital, Zhejiang University School of Medicine, Hangzhou, China

**Keywords:** IL-23 inhibitors, ulcerative colitis, meta-analysis, clinical remission, randomized controlled trials, efficacy and safety

## Abstract

**Background:**

Interleukin-23 (IL-23) plays a central role in the pathogenesis of ulcerative colitis (UC) by promoting Th17-mediated intestinal inflammation. While monoclonal antibodies targeting the IL-23p19 subunit have shown promise in clinical trials, the overall efficacy and safety of IL-23 inhibitors in moderate to severe UC remain to be comprehensively quantified.

**Methods:**

We conducted a systematic review and meta-analysis of randomized controlled trials (RCTs) evaluating the efficacy and safety of IL-23p19 inhibitors—including mirikizumab, guselkumab, and risankizumab—in adults with moderate to severe UC. A comprehensive literature search was performed in PubMed, Embase, Web of Science, and Cochrane Library through February 2025. Primary outcomes included clinical remission and clinical response during induction; secondary outcomes included endoscopic remission and adverse events. Risk ratios (RRs) and 95% confidence intervals (CIs) were calculated using a random-effects model.

**Results:**

Six RCTs encompassing 3,640 patients were included. IL-23 inhibitors significantly improved clinical remission during induction compared to placebo (RR = 2.45; 95% CI: 2.07–2.90; *p* < 0.001; *I*^2^ = 0%), as well as clinical response (RR = 2.03; 95% CI: 1.74–2.38; *p* < 0.001). Endoscopic improvement was also significantly higher in the IL-23 group (RR = 2.49; 95% CI: 2.03–3.06; *p* < 0.001). Subgroup analyses demonstrated consistent efficacy in biologic-naïve and treatment-refractory populations. The incidence of any adverse events was comparable between IL-23 inhibitors and placebo (RR = 0.91; 95% CI: 0.85–0.98; *p* = 0.01), with no increase in serious infections.

**Conclusion:**

IL-23p19 inhibitors are effective and well tolerated in inducing and maintaining remission in patients with moderate to severe UC, including those with prior treatment failure. These findings support their use as a valuable therapeutic option in the evolving management landscape of UC.

## 1 Introduction

Ulcerative colitis (UC) is a chronic, relapsing–remitting inflammatory bowel disease (IBD) characterized by continuous mucosal inflammation of the rectum and colon (1). The exact etiology remains unclear, but it is widely believed to involve a complex interplay of genetic, environmental, microbial, and immune factors. Clinically, UC presents with persistent or recurrent episodes of bloody diarrhea, abdominal pain, urgency, and weight loss, and can lead to serious complications such as toxic megacolon, perforation, and colorectal cancer (2, 3). The disease exerts a substantial burden on patients’ quality of life and healthcare systems globally (4). In the United States alone, the annual direct medical costs per UC patient range from $6,200 to $11,400, with the overall economic burden reaching up to $14.9 billion annually.

Currently, the incidence and prevalence of UC continue to rise globally, not only in traditionally high-burden regions such as Europe and North America but also increasingly in newly industrialized and developing countries, reflecting a shift in disease burden worldwide (5–6). According to recent epidemiological estimates, more than 5 million individuals are affected by UC across the globe, with over 35% of patients presenting with moderate to severe disease at initial diagnosis (7). This growing burden underscores an urgent need for more effective and safer therapeutic strategies, especially for those with moderate to severe UC who are at greater risk for complications and treatment failure.

The primary goals of UC treatment are to induce and maintain clinical and endoscopic remission, prevent disease progression, and improve long-term outcomes (8). Current pharmacologic options include aminosalicylates, corticosteroids, immunosuppressants, biologics, and small-molecule targeted agents (9). However, limitations in efficacy and safety of these therapies persist. For example, up to 32.7% of patients do not respond to 5-aminosalicylic acid (5-ASA), while prolonged use of high-dose 5-ASA is associated with nephrotoxicity (10). Corticosteroids are effective in the short term but are linked to substantial adverse effects and steroid dependency (11). Traditional immunosuppressants such as azathioprine have delayed onset of action, modest remission rates, and increased risks of bone marrow suppression and lymphoma (12). Despite the availability of anti–tumor necrosis factor (TNF) agents and anti-integrins, primary nonresponse rates remain as high as 31.4%, and secondary loss of response occurs in up to 15.7% of patients annually (13).

Recent advances have identified interleukin-23 (IL-23), a heterodimeric cytokine composed of p19 and p40 subunits, as a key driver of intestinal inflammation in UC (14). Elevated expression of IL-23 and its receptor (IL-23R) has been observed in the colonic mucosa of UC patients and correlates with disease severity (15). IL-23 promotes the differentiation and expansion of Th17 cells *via* activation of the JAK–STAT signaling pathway, leading to increased secretion of proinflammatory cytokines such as IL-17 and IL-22, and suppresses anti-inflammatory IL-10 production by lamina propria monocytes (16). It also supports the expansion of memory T cells, perpetuating chronic intestinal inflammation (17).

Monoclonal antibodies selectively targeting the IL-23p19 subunit, including mirikizumab, risankizumab, and guselkumab, have demonstrated promising efficacy and safety profiles in recent phase II and III trials (18). These agents offer a potentially more targeted therapeutic approach with a favorable risk–benefit balance. However, head-to-head comparisons are lacking, and variability in patient populations, trial designs, and outcome definitions introduces heterogeneity across studies. Furthermore, subgroup data, especially in patients refractory to anti-TNF agents or Janus kinase inhibitors, remain limited, hindering personalized treatment strategies.

To address these knowledge gaps, we conducted a comprehensive meta-analysis of randomized controlled trials (RCTs) to evaluate the efficacy and safety of IL-23p19 inhibitors in patients with moderate to severe UC. By synthesizing the most up-to-date clinical trial evidence, this study aims to provide robust, evidence-based guidance for clinicians and inform therapeutic decision-making in this evolving landscape.

## 2 Methods

### 2.1 Search strategy

A thorough literature search was conducted across multiple databases, including PubMed, EMBASE, the Cochrane Library, and Web of Science, spanning from their inception to February 2025. Considering the need for higher accuracy and clinical guidance, retrieved literature is restricted to RCTs. Search terms incorporated subject headings alongside free-text terms, including but not limited to the following: ((Idiopathic Proctocolitis) OR (Inflammatory Bowel Disease, Ulcerative Colitis Type) OR (Ulcerative Colitis) OR (Colitis Gravis)) AND ((Risankizumab) OR (Mirikizumab) OR (Guselkumab) OR (Briakinumab) OR (Interleukin 23) OR (IL - 23)). An institutional librarian proficient in systematic reviews, together with two authors, M. He and H. Liang, undertook this search.

### 2.2 Eligibility criteria and study selection

Throughout the article selection and exclusion process, rigorous adherence to the “PICOS” principle was ensured. Each data item of “PICOS” was independently and meticulously recorded in Excel by two authors. The detailed inclusion criteria are as follows: (a) The study population comprised patients aged 18 years or older; (b) The intervention involved IL-23 inhibitors, with comparisons made against placebo and/or other treatments; (c) The outcomes assessed included clinical remission, clinical response, and drug-related adverse events; (d) The study encompassed Phase II/II RCTs. Studies that did not meet any of the following criteria were excluded: (a) the article was not written in English; (b) the study data were either unextractable or missing; (c) the use of biologics other than IL-23 inhibitors in the treatment; (d) for studies derived from the same participants, only the most recent study would be evaluated.

Two reviewers (A and B) conducted an initial screening of articles meeting the inclusion criteria by perusing the abstracts. After removing duplicate entries, the articles demanding full-text scrutiny are determined, and a more in-depth verification is carried out in accordance with the PICOS principle. Each included article’s full text was comprehensively reviewed. Any disagreements during screening or full-text review were resolved by the senior author.

### 2.3 Data extraction

Data extraction encompassed patients’ baseline characteristics, primary outcomes, secondary outcomes, adverse events, and other outcome metrics of interest. Two authors utilized a pre-defined table table in Excel to record the information, and any contentious content was resolved via discussion. The characteristics of the extracted studies were as follows: author, publication year, country, trial phase, characteristics of participants (such as age, gender, disease duration, prior experience with biological therapies, etc.), number of patients, treatment regimens, follow - up duration, and reported outcomes. The primary outcome of the induction therapy was the attainment of clinical remission, which was defined according to the modified Mayo score. Secondary endpoints encompassed the achievement of clinical response, endoscopic improvement and normalization, histo-endoscopic mucosal improvement, and symptomatic remission (Table 1).

**Table 1:** Characteristics of included RCTs.

Author, Year	Centers, *n*	Phase	Setting	Sample size, *n*	UC severity	Therapeutic schedule	Duration of induction phase	Primary outcome
Peyrin-Biroulet et al. (2023) (19)	141 (27 countries)	2b	Multicenter, double-blinded, RTC	313	Moderate-to-severe active UC	IV guselkumab 200/400 mg at week 0, 4, 8	12 weeks	Clinical response at week 12
D’Haens et al (2023) (20)	383(34 countries)	3	Multicenter, double-blinded, RTC	1162	Moderate-to-severe active UC	IV mirikizumab 300 mg at week 0, 4, 8	12 weeks	Clinical response at week 12
Rubin et al. (2025) (21) (QUASAR)	254(32 countries)	3	Multicenter, double-blinded, RTC	701	Moderate-to-severe active UC	IV guselkumab 200 mg at week 0, 4, 8	12 weeks	Clinical response at week 12
Sandborn et al. (2020) (22)	75(14 countries)	2	Multicenter, double-blinded, RTC	249	Moderate-to-severe active UC	IV mirikizumab EB 50/EB 200/ 600 mg at week 0, 4, 8	12 weeks	Clinical response at week 12
Louis et al. (2024) (23) (INSPIRE)	261(41 countries)	3	Multicenter, double-blinded, RTC	975	Moderate-to-severe active UC	IV risankizumab 1,200 mg at week 0, 4, 8	12 weeks	Clinical response at week 12
Louis et al. (2024) (23)	NA	2b	Multicenter, double-blinded, RTC	240	Moderate-to-severe active UC	IV risankizumab 600/1200/1800 mg at week 0, 4, 8	12 weeks	Clinical response at week 12

### 2.4 Quality assessment

According to the guidelines presented in the Cochrane Handbook for Systematic Reviews of Interventions, the bias risk associated with the included RCTs was evaluated across the following domains: (1) random sequence generation, (2) allocation concealment, (3) blinding of participants and research personnel, (4) blinding of outcome assessment, (5) incomplete outcome data, (6) selective outcome reporting, and (7) other potential sources of bias. For each domain, the data extractors classified the included trials into one of three categories: low bias risk, unclear bias risk, or high bias risk. A and B independently assessed each domain for all articles. Any discrepancies were resolved through comprehensive discussion (Table 2).

**Table 2:** Characteristics of patients and UC at baseline.

Study, Year	Group	No. of patients	Age, mean (SD) y	Female, No. (%)	Duration of UC, mean(SD), y	Location and extent of disease, No. (%)	
						Left-sided colitis	Extensive
Peyrin-Biroulet et al. (2023) (19) (QUASAR)	GUS (200 mg)	101	43.3 (14.28)	41 (40.6)	7.0 (6.00)	NA	48 (47.5)
	GUS (400 mg)	107	40.4 (13.84)	48 (44.9)	7.9 (7.15)	NA	59 (55.1)
	Placebo	105	41.2 (15.05)	39 (37.1)	7.7 (7.16)	NA	46 (43.8)
D’Haens et al. (2023) (20) (Lucent)	MIR (300 mg)	868	42.9 (13.9)	338 (38.9)	7.2 (6.7)	544 (62.7)	NA
	Placebo	294	41.3 (13.8)	129 (43.9)	6.9 (7.0)	188 (64.2)	NA
Rubin et al. (2025) (21) (QUASAR)	GUS (200 mg)	421	41.0 (13.9)	183 (43)	7.8 (7.7)	NA	188 (45)
	Placebo	280	39.8 (13.4)	119 (42)	7.1 (6.5)	NA	147 (52)
Sandborn et al. (2020) (22)	MIR EB 50 mg	63	41.8 (14.1)	25 (39.7)	8.2 (7.2)	NA	NA
	MIR EB 200 mg	62	43.4 (4.7)	25 (40.3)	9.0 (9.0)	NA	NA
	MIR 600 mg	61	42.4 (13.4)	23 (37.7)	6.0 (5.7)	NA	NA
	Placebo	63	42.6 (13.5)	27 (42.9)	9.5 (9.6)	NA	NA
Louis et al. (2024) (23) (INSPIRE)	RIS 1200 mg	650	41.8 (13.5)	265 (40.8)	7.7 (6.9)	313 (48.2)	334 (51.4)
	Placebo	325	42.8 (14.3)	124 (38.2)	8.1 (7.0)	150 (46.2)	174 (53.5)
Louis et al. (2024) (23)	RIS 600 mg	61	43.0 (14.9)	21 (34.4)	10.0 (6.9)	29 (47.5)	32 (52.5)
	RIS 1200 mg	61	41.8 (13.8)	26 (42.6)	9.8 (8.3)	22 (36.1)	39 (63.9)
	RIS 1800 mg	58	40.9 (13.7)	27 (46.6)	8.5 (5.5)	23 (39.7)	35 (60.3)
	Placebo	60	44.4 (14.1)	36 (60.0)	10.4 (6.8)	30 (50.0)	30 (50.0)

Study, Year	Extraintestinal manifestation present, No. (%)	MMS* mean (SD)	ES** of 3, No. (%)	CRP*** median (IQR), mg/L	Fecal calprotectin median (IQR), mg/kg
Peyrin-Biroulet et al. (2023) (19) (QUASAR)	15 (14.9)	7.0 (1.06)	66 (65.3)	4.3 (1.6–17.8)	1667.0 (771.0–2859.0)
	22 (20.6)	7.0 (0.99)	78 (72.9)	4.4 (1.9–8.8)	1578.0 (811.0–2860.0)
	13 (12.4)	6.9 (1.06)	75 (71.4)	4.9 (1.4–10.8)	1457.0 (749.0–3054.0)
D’Haens et al. (2023) (20) (Lucent)	NA	NA	574 (66.1)	4.1 (1.5–9.6)	1559.0 (634.0–3210.0)
	NA	NA	200 (68.3)	4.2 (1.2–9.5)	1471.5 (626.5–2944.5)
Rubin et al. (2025) (21) (QUASAR)	60 (14)	6.9 (1.1)	296 (70)	4.3 (1.5–11.2)	1651.0 (647.0–3479.0)
	30 (11)	6.9 (1.1)	180 (64)	3.8 (1.6–9.1)	1606.0 (654.0–3077.0)
Sandborn et al. (2020) (22)	NA	6.6 (1.3)	50 (79.4)	4.5 (1.4–12.6)	1945 (510–2,992)
	NA	6.4 (1.4)	41 (66.1)	3.6 (1.4–13.7)	1,560 (399–2,443)
	NA	6.5 (1.3)	45 (73.8)	6.2 (2.0–15.5)	1,523 (619–2,587)
	NA	6.7 (1.2)	47 (74.6)	3.9 (1.1–11.2)	1,353 (618–2,481)
Louis et al. (2024) (23) (INSPIRE)	NA	7.1 (1.2)	442 (68.0)	3.4 (1.2–8.6)	1530.0 (592.0–3196.0)
	NA	7.1 (1.3)	231 (71.1)	4.0 (1.2–9.5)	1624.0 (601.0–3493.0)
Louis et al. (2024) (23)	NA	6.9 (1.2)	NA	3.3	1792
	NA	7.0 (1.1)	NA	6.4	1,436
	NA	7.2 (1.4)	NA	4.7	1,467
	NA	7.0 (1.2)	NA	3.4	1750

Study, Year	Baseline therapy for ulcerative colitis, No. (%)			History of inadequate response or intolerance to a biologic or JAK inhibitor
	Oral corticosteroids	Immunosuppressants	Oral aminosalicylates	
Peyrin-Biroulet et al. (2023) (19) (QUASAR)	41 (40.6)	25 (24.8)	74 (73.3)	46 (45.5)
	44 (41.1)	27 (25.2)	89 (83.2)	51 (47.7)
	40 (38.1)	17 (16.2)	79 (75.2)	51 (48.6)
D’Haens et al. (2023) (20) (Lucent)	351 (40.4)	211 (24.3)	646 (74.4)	361 (41.6)
	113 (38.4)	69 (23.5)	217 (73.8)	118 (40.1)
Rubin et al. (2025) (21) (QUASAR)	182 (50)	92 (25)	304 (83)	208 (49.0)
	120 (49)	54 (22)	204 (83.0)	136 (49.0)
Sandborn et al. (2020) (22)	29 (46.0)	14 (22.2)	41 (65.1)	NA
	25 (40.3)	18 (29.0)	56 (90.3)	NA
	34 (55.7)	11 (18.0)	39 (63.9)	NA
	33 (52.4)	25 (39.7)	47 (74.6)	NA
Louis et al. (2024) (23) (INSPIRE)	236 (36.3)	108 (16.6)	475 (73.1)	317 (48.8)
	112 (34.5)	53 (16.3)	238 (73.2)	155 (47.7)
Louis et al. (2024) (23)	27 (44.3)	14 (23.0)	33 (54.1)	61 (100)
	30 (49.2)	15 (24.6)	40 (65.6)	61 (100)
	25 (43.1)	11 (19.0)	42 (72.4)	58 (100)
	25 (41.7)	10 (16.7)	38 (63.3)	60 (100)

*MMS: Modified Mayo score; **ES: endoscopic subscore; ***CRP: C-reactive protein.

### 2.5 Statistical analysis

In this meta-analysis, the RevMan 5.4 software was selected to conduct statistical analysis. The risk ratio (RR) was used as the key indicator of the effect size, and its 95% confidence interval (CI) was calculated to evaluate the effectiveness of the intervention measures. Given the intricate and varied baseline characteristics of patients, coupled with the extensive range of IL-23 inhibitors, the DerSimonian and Laird random-effects model for multiple outcomes was opted to analyze the outcomes of the included studies. This model is capable of comprehensively accounting for the diverse differential factors present among the studies. Moreover, the *I*^2^ statistic was used for evaluation. When the *I*^2^ value is 0%, there is no obvious heterogeneity in the included studies, and the results of each study are highly consistent; an increase in the *I*^2^ value indicates an enhancement of heterogeneity. If the number of included studies exceeds 10, publication bias analysis will be conducted through Egger’s weighted regression test to identify potential biases caused by publication bias. When fewer than 10 clinical studies were available, we assessed potential publication bias for the primary endpoint (clinical remission during induction) using a funnel plot. If *I*^2^ values are substantial, sensitivity analyses are performed to pinpoint the factors contributing to heterogeneity. In all of the aforementioned statistical analyses, a *p* value of <0.05 was established as the threshold for determining statistical significance.

## 3 Results

### 3.1 Search results and study characteristics

**Figure 1:**
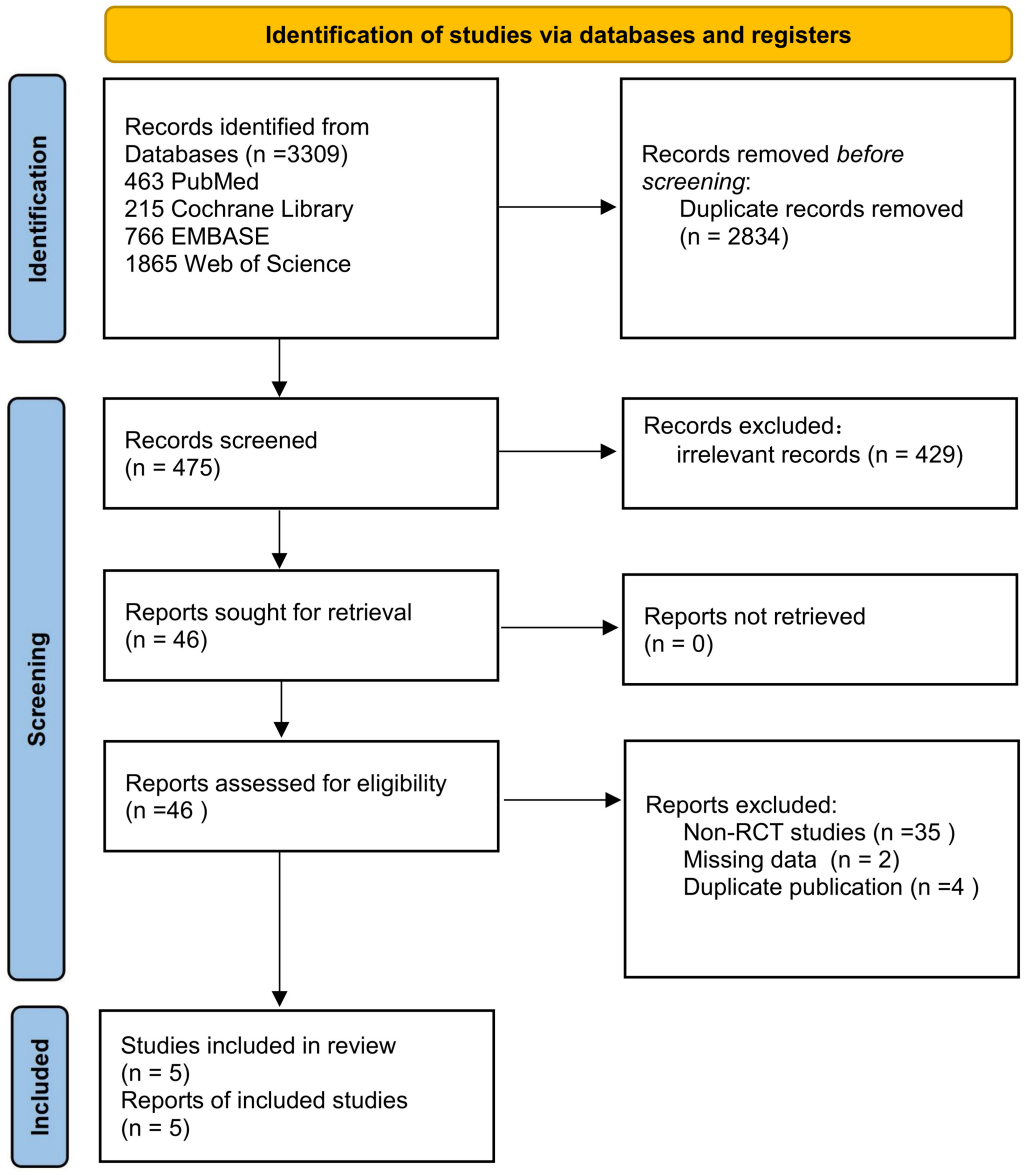
PRISMA flow diagram of the study selection process. Flow chart showing the identification, screening, eligibility assessment, and inclusion of RCTs evaluating IL-23 inhibitors in moderate to severe ulcerative colitis, in accordance with the PRISMA 2020 guidelines.

A total of 3,309 records were initially retrieved from PubMed, Embase, Web of Science, and the Cochrane Library. After removing duplicates using EndNote X9, 475 records remained. Title and abstract screening excluded irrelevant studies, resulting in 46 full-text articles for further review. Ultimately, six RCTs reported in five articles met all inclusion criteria and were included in this meta-analysis (19–23). All included studies were multicenter, multinational, and double-blind RCTs. The study selection process is illustrated in the PRISMA flow diagram (Figure 1).

The 6 included studies collectively enrolled 3,640 patients with moderate to severe ulcerative colitis. Among them, two trials evaluated mirikizumab, two assessed guselkumab, and two examined risankizumab. One of the risankizumab studies included a dedicated Phase 2b induction sub-study designed to identify optimal induction dosing. The mean age of the included patients with moderate to severe ulcerative colitis ranged from 40.2 to 42.5 years, with females accounting for 39.1 to 43.1% of the participants. The average duration of the disease was between 7.1 and 9.7 years. All included studies used a placebo control, and all studies reported safety outcomes and the clinical remission rate at week 12. Detailed study characteristics and baseline demographics are summarized in Table 1, 2.

### 3.2 Quality assessment

All included studies were randomized, double-blind, multicenter trials. Using the Cochrane Risk of Bias tool, the overall methodological quality was deemed high, with low risk of bias across key domains, including random sequence generation, allocation concealment, blinding, and outcome reporting. A visual summary of the bias assessment is presented in Supplementary Figures 1, 2. Given the limited number of trials contributing to the primary endpoint, the statistical power of Egger’s regression would be markedly reduced; therefore, we did not perform Egger’s test. Instead, we generated a funnel plot to qualitatively assess potential small-study effects/publication bias. The funnel plot for the primary outcome (clinical remission during induction) is presented as Supplementary Figures 3. Visual inspection did not reveal marked asymmetry.

### 3.3 Efficacy analysis

**Figure 2:**
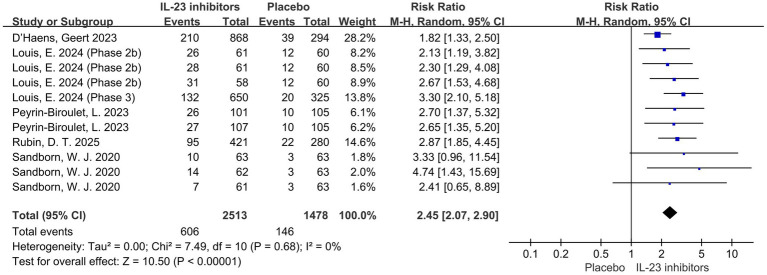
Forest plot of clinical remission during induction phase. Pooled analysis of RCTs evaluating the effect of IL-23p19 inhibitors versus placebo on clinical remission rates during the induction phase in patients with moderate to severe ulcerative colitis.

All studies reported the primary outcome of clinical remission. Pooled data from six trials demonstrated that IL-23 inhibitors significantly improved clinical remission rates during the induction phase compared to placebo (RR = 2.45; 95% CI: 2.07–2.90; *p* < 0.001; *I*^2^ = 0%) (Figure 2).

**Figure 3:**
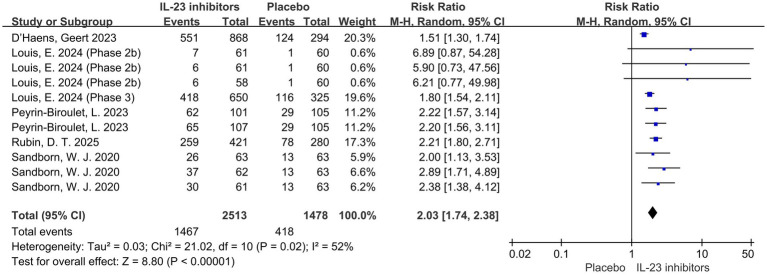
Forest plot of clinical response during induction phase. Comparison of clinical response rates between IL-23p19 inhibitors and placebo during induction therapy.

Subsequently, the included studies were analyzed with respect to secondary outcomes. Compared with the control group, a significant improvement in clinical response was also observed with IL-23 inhibitors during induction therapy (RR = 2.03; 95% CI: 1.74–2.38; *p* < 0.001; *I*^2^ = 52%) (Figure 3).

**Figure 4:**
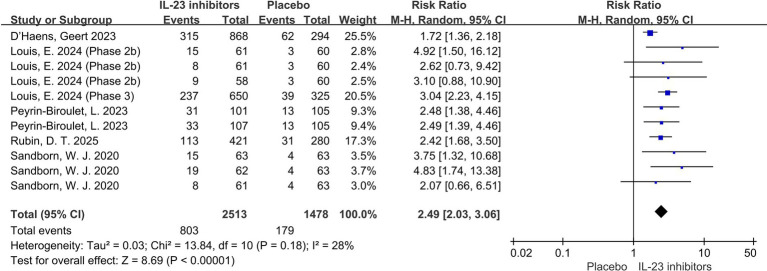
Forest plot of endoscopic improvement during induction phase. Meta-analysis of the effect of IL-23 inhibitors on achieving endoscopic improvement compared with placebo during the induction phase.

**Figure 5:**
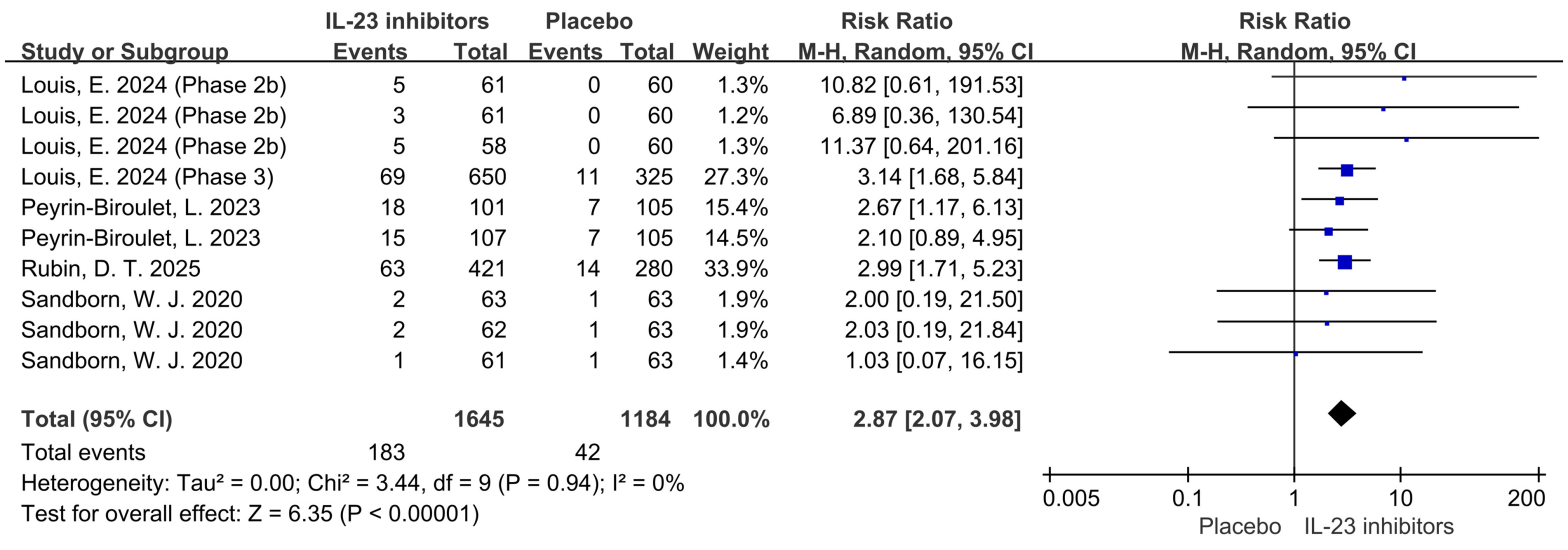
Forest plot of endoscopic normalization during induction phase. Comparison of the effect of IL-23 inhibitors versus placebo on achieving endoscopic normalization during the induction phase.

Endoscopic findings are also one of the key indicators for evaluating treatment efficacy. Compared to the placebo group, the IL-23 inhibitor group had a significantly higher likelihood of achieving endoscopic improvement (RR = 2.49; 95% CI: 2.03–3.06; *p* < 0.001; *I*^2^ = 28%) and endoscopic normalization (RR = 2.87; 95% CI: 2.07–3.98; *p* < 0.001; *I*^2^ = 0%) during the 12-week induction phase (Figures 4, 5). Moreover, a higher proportion of patients in the IL-23 inhibitor group achieved the histo-endoscopic mucosal improvement criteria compared to the control group (RR = 2.60; 95% CI: 2.04–3.31; *p* < 0.001; *I*^2^ = 26%) (Supplementary Figures 4). These findings suggest that IL-23 inhibitors are more effective in improving both histological and endoscopic mucosal healing compared to placebo. In addition, rates of symptomatic remission were consistently higher in patients receiving IL-23 inhibitors compared with placebo during induction phase (RR = 1.96; 95% CI: 1.53–2.52; *p* < 0.001; *I*^2^ = 58%) (Supplementary Figures 5), further supporting the symptomatic efficacy of these agents.

### 3.4 Subgroup analyses

To further explore the factors influencing treatment outcomes, we conducted subgroup analyses to evaluate the potential benefits of IL-23 inhibitors in populations with different prior medication histories, which may provide important insights for clinical treatment decisions.

**Figure 6:**
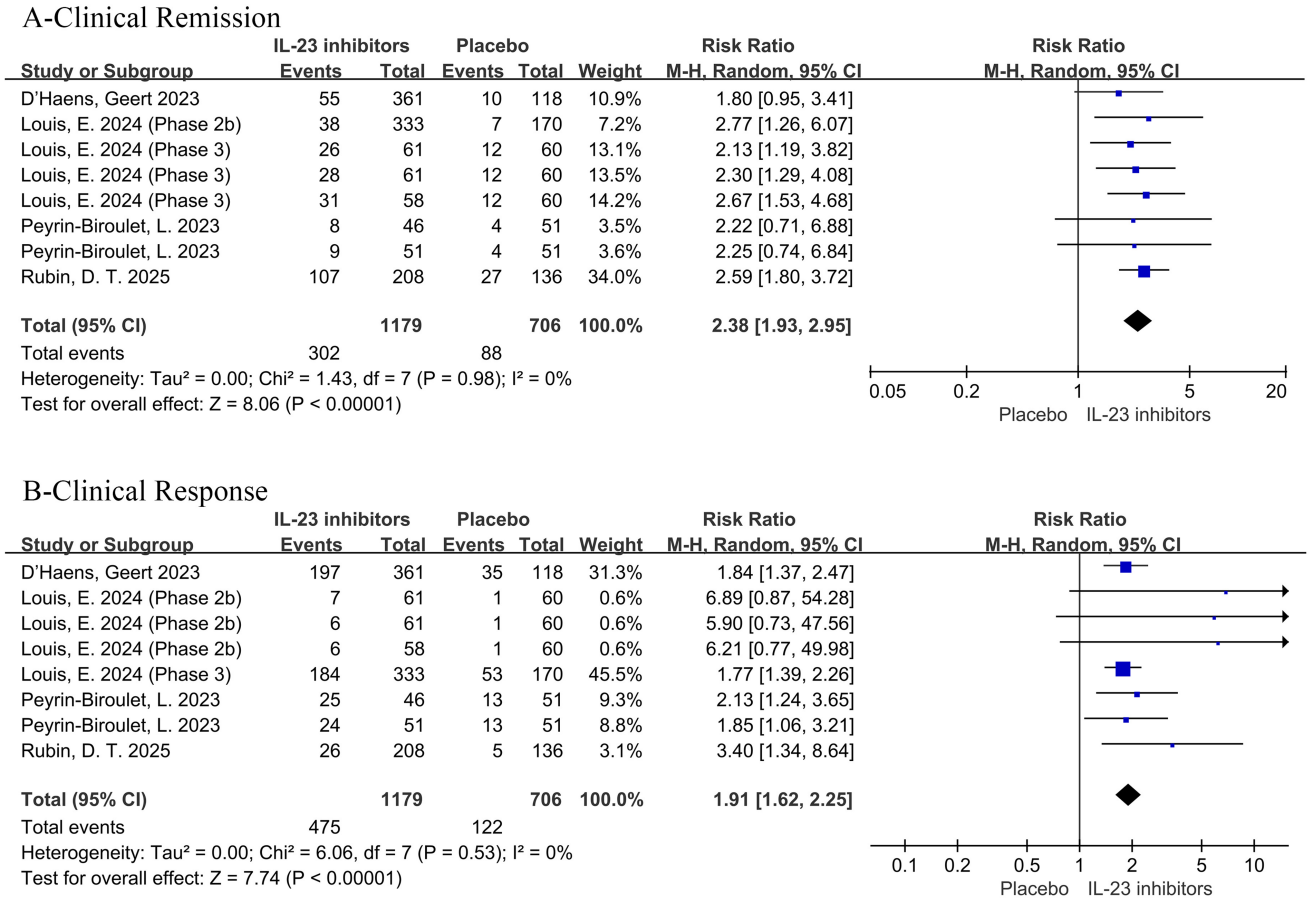
Forest plot of efficacy outcomes in the subgroup of patients with prior inadequate response or intolerance to biologics or JAK inhibitors. Outcomes include **(A)** clinical remission and **(B)** clinical response to IL-23 inhibitors versus placebo during induction therapy.

**Figure 7:**
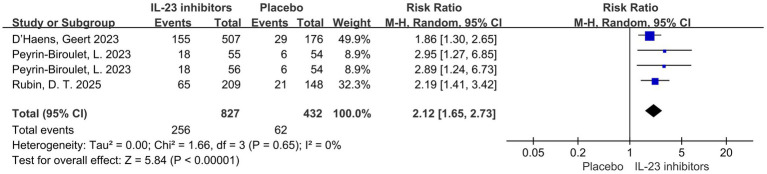
Forest plot of clinical remission in patients without prior inadequate response or intolerance to biologics or JAK inhibitors. Comparison of clinical remission rates between IL-23 inhibitors and placebo during the induction phase.

Regarding prior treatment, all patients had received conventional therapy, while 50.5% of participants had a history of inadequate response or intolerance to biologics or JAK inhibitors. Patients were stratified based on the presence or absence of such a history. The association The association between IL-23 inhibitors and placebo remained consistent across both subgroups. Among those with prior inadequate response or intolerance to biologics or JAK inhibitors, the RR were as follows: clinical remission (RR = 2.38, 95% CI = 1.93–2.95, *p* < 0.001), clinical response (RR = 1.91, 95% CI = 1.62–2.25, *p* < 0.001), endoscopic improvement (RR = 2.18, 95% CI = 1.52–3.13, *p* < 0.001), endoscopic normalization (RR = 1.95 95% CI = 1.05–3.59, *p* = 0.03), symptomatic remission (RR = 2.29, 95% CI = 1.78–2.96, *p* < 0.001) and histo-endoscopic mucosal improvement (RR = 2.48, 95% CI = 1.71–3.58, *p* < 0.001) (Figure 6; Supplementary Figures 6). In patients with inadequate response or intolerance to previous biologics or JAK inhibitors, IL-23 inhibitors still demonstrated favorable clinical remission and response outcomes. This suggests that IL-23p19 blockade may serve as a viable option in treatment-refractory UC populations. Similarly, among patients without a prior history of inadequate response or intolerance to biologics or JAK inhibitors, the RR for clinical remission was 2.12 (95% CI = 1.65–2.73, *p* < 0.001) (Figure 7).

**Figure 8:**
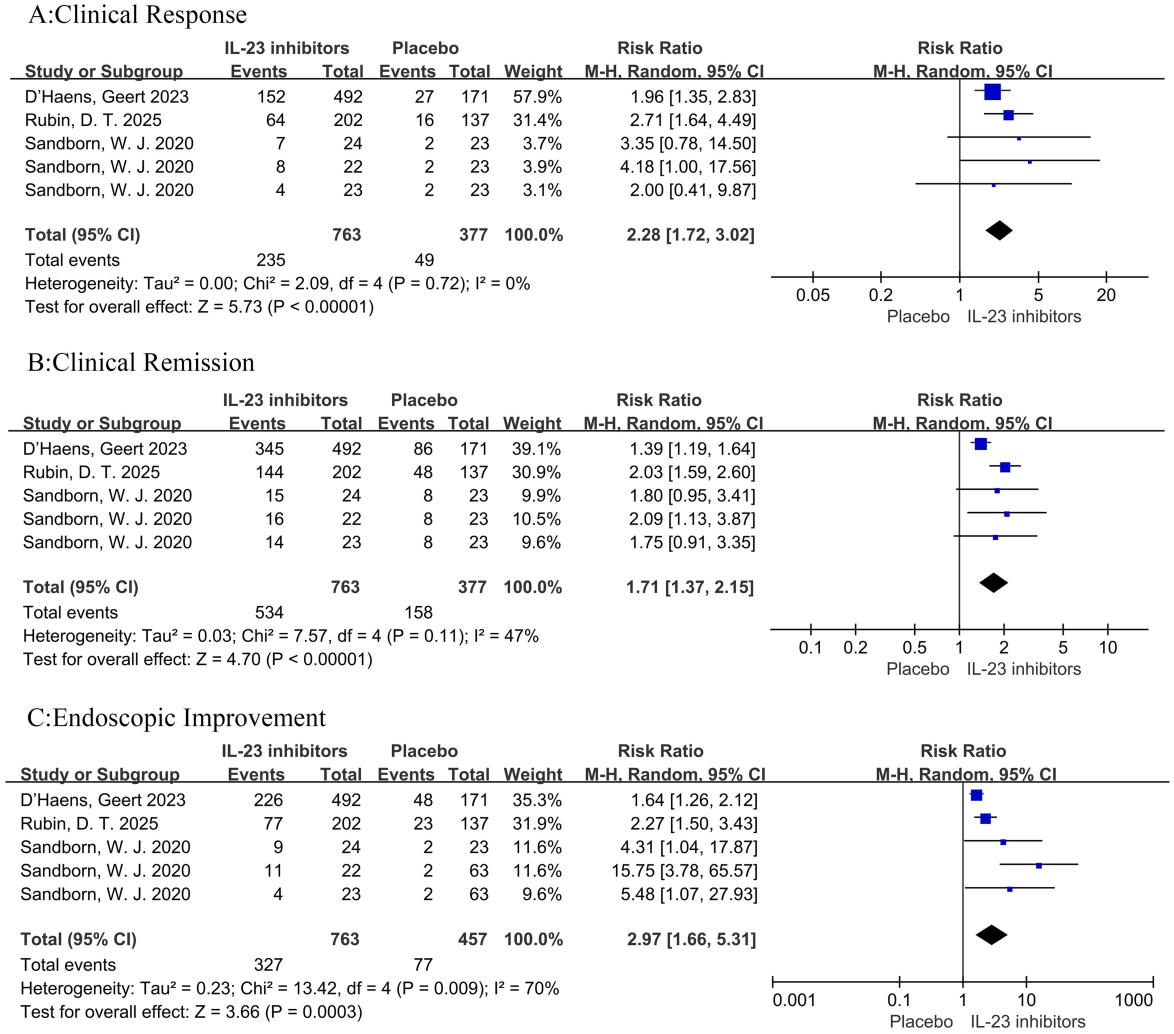
Forest plot of subgroup analysis for patients naive to biologics or JAK inhibitors. Comparison of the effect of IL-23 inhibitors versus placebo on **(A)** clinical remission, **(B)** clinical response, and **(C)** endoscopic improvement during induction therapy.

In addition, subgroup analysis revealed that compared with placebo among patients who had not previously received biologics or JAK inhibitors, treatment with IL-23 inhibitors was associated with higher rates of clinical remission (RR = 2.28, 95% CI = 1.72–3.02, *p* < 0.001), clinical response (RR = 1.71, 95% CI = 1.37–2.15, *p* < 0.001), and endoscopic improvement (RR = 2.97, 95% CI = 1.66–5.31, *p* = 0.009) (Figure 8).

### 3.5 Safety outcomes

Among the 2,603 participants who received IL-23 inhibitor treatment, 1,197 (46.0%) reported adverse events, including anemia, infections, nasopharyngitis, and arthralgia. Similarly, adverse events were reported in the placebo group, with 575 of 1,154 participants (49.8%) affected.

**Figure 9:**
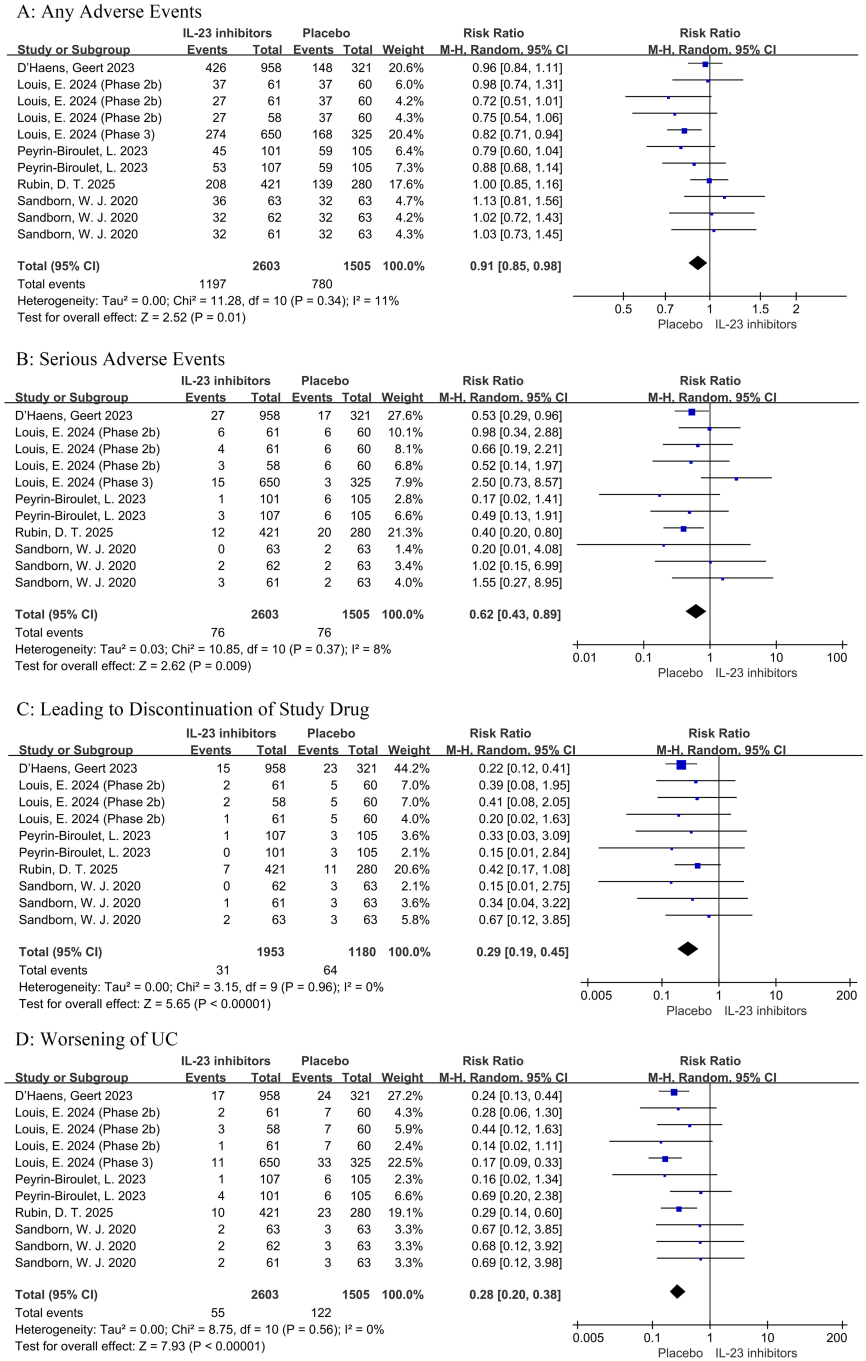
Forest plot of safety outcomes during induction phase. Comparison of the effect of IL-23 inhibitors versus placebo on **(A)** any adverse events, **(B)** serious adverse events, **(C)** adverse events leading to treatment discontinuation, and **(D)** worsening of ulcerative colitis.

During the induction phase, IL-23 inhibitors were associated with a significantly lower risk of any adverse events (RR = 0.91, 95% CI: 0.85–0.98, *p* < 0.05) and serious adverse events (RR = 0.62, 95% CI: 0.43–0.89, *p* < 0.05) compared with placebo (Figure 9). Furthermore, the IL-23 inhibitor group showed significantly reduced risks of adverse events leading to treatment discontinuation (RR = 0.29, 95% CI: 0.19–0.45, *p* < 0.001) and worsening of ulcerative colitis (RR = 0.28, 95% CI: 0.20–0.38, *p* < 0.001). In addition, meta-analysis of treatment-related adverse events revealed no significant differences between IL-23 inhibitors and placebo with respect to the serious infection (Supplementary Figures 7). Collectively, these findings suggest that IL-23 inhibitors exhibit a favorable safety profile.

## 4 Discussion

Ulcerative colitis (UC) is a chronic, relapsing inflammatory disorder that substantially impairs patients’ quality of life and confers a long-term risk of colorectal cancer (24, 25). Although the introduction of biologic agents, particularly tumor necrosis factor (TNF) antagonists, has transformed the therapeutic landscape of UC over the past two decades, significant limitations remain in clinical practice, with primary nonresponse rates approaching 30% and a progressive increase in secondary loss of response over time (26, 27). Consequently, the development of novel agents with distinct mechanisms of action has become a major focus in the management of inflammatory bowel disease in recent years.

The interleukin-23 (IL-23)/Th17 pathway plays a central role in the immunopathogenesis of ulcerative colitis (UC). Secreted by dendritic cells and macrophages, IL-23 promotes the expansion and maintenance of Th17 cells, which in turn drive the release of multiple proinflammatory cytokines such as IL-17 and IL-22, thereby sustaining chronic mucosal inflammation (28). Genetic studies further support the pivotal involvement of IL-23 in disease pathogenesis, demonstrating that variants in the IL23R gene are significantly associated with susceptibility to inflammatory bowel disease (IBD). Certain loss-of-function variants have been shown to attenuate inflammatory responses and confer protection against disease development (29–31).

Consistent with the immunopathogenic role of the IL-23/Th17 axis in UC, selective blockade of the p19 subunit has emerged as a promising strategy targeting upstream inflammatory mediators while sparing IL-12–related pathways, potentially minimizing systemic immunosuppression (14, 18). With advances in the understanding of the IL-23 signaling pathway, monoclonal antibodies specifically targeting the IL-23 p19 subunit—such as risankizumab, guselkumab, and mirikizumab—have been developed and progressively introduced into clinical practice. Compared with earlier agents that simultaneously block the shared IL-12/23 p40 subunit (e.g., ustekinumab), IL-23 inhibitors offer greater selectivity, potentially preserving therapeutic efficacy while reducing non-specific immunosuppression, which may translate into a more favorable safety profile. Recent RCTs have demonstrated that IL-23 inhibitors significantly improve clinical remission and mucosal healing rates in patients with moderate-to-severe UC, along with exhibiting a well-tolerated safety profile.

This meta-analysis provides comprehensive evidence supporting the efficacy and safety of IL-23p19 inhibitors—namely mirikizumab, guselkumab, and risankizumab—in the treatment of moderate to severe ulcerative colitis (UC). Our pooled analysis demonstrated that IL-23 inhibitors significantly improved clinical remission, clinical response, and endoscopic outcomes during induction phase compared with placebo. Notably, the favorable safety profile of IL-23 inhibitors further reinforces their potential as effective therapeutic agents for UC, including in treatment-refractory populations.

In our analysis, IL-23 inhibitors yielded a > 2-fold increase in clinical remission during induction (RR = 2.45), with low heterogeneity (*I*^2^ = 0%), suggesting a consistent effect across different agents and trial designs. Furthermore, clinical response improvements were robust (RR = 2.03), and endoscopic improvement was significantly enhanced (RR = 2.49), highlighting not only symptomatic benefit but also mucosal healing—an established predictor of sustained remission and reduced long-term complications (8).

Subgroup analyses were conducted in this study to further evaluate the efficacy of IL-23 inhibitors in patients with ulcerative colitis (UC) stratified by prior treatment history. The results demonstrated that IL-23 inhibitors conferred significant and consistent benefits over placebo, irrespective of previous inadequate response or intolerance to biologics or JAK inhibitors. In patients with prior inadequate response or intolerance to biologics or JAK inhibitors, IL-23 inhibitors showed significantly higher efficacy across multiple key endpoints, including clinical remission, clinical response, endoscopic improvement, endoscopic normalization, symptomatic remission, and histo-endoscopic mucosal improvement (all RR > 1.9, *p* < 0.05). These findings suggest that IL-23p19 blockade represents an effective therapeutic option even in treatment-refractory UC populations. Similarly, in patients without such prior treatment failure, IL-23 inhibitors also demonstrated significant clinical benefits. The risk ratio for clinical remission (RR = 2.12) was consistent with that observed in the overall population and the refractory subgroup, supporting the therapeutic potential of these agents in a broader UC population. Notably, among patients naive to biologics or JAK inhibitors, IL-23 inhibitors were associated with significantly higher rates of clinical remission, clinical response, and endoscopic improvement (*p* < 0.01), indicating their suitability not only for treatment-experienced populations but also as a potential first-line biologic therapy for moderate-to-severe UC.

These subgroup analyses reinforce the role of IL-23 inhibitors as effective treatments for UC, regardless of prior exposure to biologics or JAK inhibitors. The findings have important implications for clinical decision-making, particularly in offering an effective new therapeutic pathway for patients with prior treatment failure.

From a safety perspective, IL-23 inhibitors were not associated with increased adverse events (RR = 0.91) and serious infections (RR = 1.00). This favorable profile is particularly relevant in contrast to anti-TNF agents and JAK inhibitors, which carry risks of serious infections, malignancy, and thromboembolic events (13). The selective targeting of IL-23, without affecting IL-12 or interferon-*γ* signaling, may account for the lower immunosuppressive burden (16).

To situate our findings within the broader therapeutic landscape, we reviewed and synthesized recent network meta-analyses and guideline recommendations on advanced therapies. Overall, JAK inhibitors—particularly upadacitinib—ranked among the most effective or outperformed many biologics and small molecules for both induction and maintenance outcomes, including clinical remission, endoscopic improvement, and histologic healing (32, 33). The IL-23 p19 inhibitors examined in our study (risankizumab, guselkumab, mirikizumab) also performed strongly across several key endpoints; risankizumab ranked first for histologic improvement during induction, and in selected maintenance comparisons mirikizumab demonstrated superior or comparable clinical outcomes versus several anti-TNF agents and other mechanisms of action (34). These observations are consistent with the 2024 AGA living clinical guideline, which positions IL-23 antagonists and JAK inhibitors as important high- or moderate-efficacy options for both biologic-naïve and previously exposed patients (35). From a safety perspective, JAK inhibitors should be used with caution in older individuals and those with cardiovascular risk factors, whereas selective IL-23 blockade—which spares IL-12/IFN-*γ* signaling—may entail a lower systemic immunosuppressive burden.

Nevertheless, this study has several limitations. First, the number of included RCTs was limited. Because fewer than 10 studies were available, quantitative tests for publication bias were underpowered; we therefore provide only a qualitative assessment using a funnel plot, and uncertainty regarding publication bias and small-study effects remains. In addition, the sample size was insufficient to yield precise risk estimates for rare but serious adverse events (e.g., opportunistic infections, thromboembolism, malignancy), which will require longer follow-up and real-world data for validation. Second, maintenance trials varied in follow-up duration and dosing/withdrawal strategies, making direct pooling prone to methodological heterogeneity; accordingly, our conclusions primarily pertain to the induction phase, and longer follow-up beyond 12–52 weeks is needed to evaluate sustained remission and relapse. Third, although we performed subgroup analyses and most included trials were multicenter and multinational, differences across regions in healthcare systems, baseline demographics (e.g., age, sex, prior exposure to biologics or small molecules, comorbidities), and disease phenotypes may limit the generalizability of our findings to broader clinical populations.

Although our evidence supports the efficacy and safety of IL-23 p19 inhibitors during induction therapy for moderate-to-severe UC, direct head-to-head comparisons between IL-23 inhibitors and other biologic therapies are not yet available. Accordingly, head-to-head RCTs versus anti-TNF agents and JAK inhibitors are warranted to clarify comparative effectiveness and safety trade-offs. In parallel, long-term real-world studies are needed to evaluate sustained remission, relapse risk, treatment persistence, and cumulative safety. In addition, we recommend stratifying patients by biomarkers and prior treatment exposure, and conducting pharmacoeconomic evaluations to inform health-care decision making.

## 5 Conclusion

In conclusion, IL-23p19 inhibitors significantly improve clinical remission, clinical response, and endoscopic outcomes in patients with moderate to severe ulcerative colitis, with a favorable safety profile. These agents represent a valuable addition to the therapeutic arsenal for UC, especially in patients with prior biologic or JAK inhibitor failure. While further research is needed to compare IL-23 inhibitors head-to-head with other advanced therapies and to validate long-term outcomes in real-world settings, current evidence strongly supports their integration into treatment algorithms for moderate to severe UC.

## Data Availability

The raw data supporting the conclusions of this article will be made available by the authors, without undue reservation.

## Glossary

5-ASA: 5-aminosalicylic acid
CI: Confidence interval
CRP: C-reactive protein
EB: Exposure-based
ES: Endoscopic subscore
IBD: Inflammatory bowel disease
IL-23: Interleukin-23
I^2^: I-squared statistic
IV: Intravenous
JAK: Janus kinase
MMS: Mayo mucosal score
NA: Not available
NEJM: New England Journal of Medicine
PRISMA: Preferred Reporting Items for Systematic Reviews and Meta-Analyses
RCT: Randomized controlled trial
RR: Risk ratio
SD: Standard deviation
TNF: Tumor necrosis factor
UC: Ulcerative colitis
