# Correction: Efficacy and safety of IL-23 inhibitors in the treatment of moderate to severe ulcerative colitis: a meta-analysis based on randomized controlled trials

**DOI:** 10.3389/fmed.2026.1836208

**Published:** 2026-04-02

**Authors:** Minyang He, Huixian Liang, Xiangcheng Fan, Leimin Sun

**Affiliations:** 1 Department of Gastroenterology, the Fourth Affiliated Hospital of School of Medicine, and International School of Medicine, International Institutes of Medicine, Zhejiang University, Yiwu, China; 2 Tongde Hospital of Zhejiang Province Affiliated to Zhejiang Chinese Medical University (Tongde Hospital of Zhejiang Province), Hangzhou, Zhejiang, China; 3 Zhejiang Academy of Traditional Chinese Medicine, Hangzhou, Zhejiang, China; 4 Department of Gastroenterology, Sir Run Run Shaw Hospital, Zhejiang University School of Medicine, Hangzhou, China

**Keywords:** IL-23 inhibitors, ulcerative colitis, meta-analysis, clinical remission, randomized controlled trials, efficacy and safety

Affiliation 1 “Department of Gastroenterology, the Fourth Affiliated Hospital of School of Medicine, and International School of Medicine, International Institutes of Medicine, Zhejiang University, Yiwu, China” was omitted for author Leimin Sun. This affiliation has now been added for author Leimin Sun.

The order of authors in the author list of the published paper was erroneously given as:

Minyang He, Huixian Liang, Leimin Sun, and Xiangcheng Fan

The correct author list reads:

Minyang He, Huixian Liang, Xiangcheng Fan, and Leimin Sun

The order of the affiliations has been updated accordingly.

The original version of this article has been updated.

