# Supplementary material for: Efficacy and safety of IL-23 inhibitors in the treatment of moderate to severe ulcerative colitis: a meta-analysis based on randomized controlled trials
Source: Front Med (Lausanne). 2025 Dec 4;12:1724448. doi: 10.3389/fmed.2025.1724448 (PMC12711713; doi:10.3389/fmed.2025.1724448)

**Supporting Information for**

***Original Article***

**Efficacy and Safety of IL‑23 Inhibitors in the Treatment of Moderate to Severe Ulcerative Colitis: A Meta‑Analysis Based on Randomized Controlled Trial**

Minyang He^1†^, Huixian Liang^1†^, Leimin Sun^2*^, Xiangcheng Fan^3,4,*^

*^1^Department of Gastroenterology, The Fourth Affiliated Hospital of School of Medicine, and International School of Medicine, International Institutes of Medicine, Zhejiang University, Yiwu, 322000, China*

*^2^Department of Gastroenterology, Sir Run Run Shaw Hospital, Zhejiang University School of Medicine, Hangzhou, 310016, China*

*^3^Department of Pharmacy, Center for Membrane Receptor and Brain Medicine, The Fourth Affiliated Hospital of School of Medicine, and International School of Medicine, International Institutes of Medicine, Zhejiang University, Yiwu, 322000, China*

*^4^Center for Innovative Traditional Chinese Medicine Target and New Drug Research, International Institutes of Medicine, Zhejiang University, Yiwu, 322000, China*

^†^These authors contributed equally.

*Correspondence: [sunlm@zju.edu.cn](mailto:sunlm@zju.edu.cn) (L.S.), and cpufxc@163.com (X.F.)

**Supplementary Figure Legends**

**Supplementary Figure 1.** **Risk of bias graph.** Summary of risk of bias for each domain across all included randomized controlled trials, expressed as percentages.

**Supplementary Figure 2.** **Risk of bias summary.** Detailed risk of bias assessment for each included randomized controlled trial using the Cochrane Risk of Bias tool.

**Supplementary Figure 3. Funnel plot for the primary outcome.** Funnel plot of clinical remission during induction across included randomized controlled trials; each point denotes a study-specific log risk ratio plotted against its standard error

**Supplementary Figure 4. Forest plot of histologic-endoscopic mucosal improvement during induction phase.** Comparison of the effect of IL-23 inhibitors versus placebo on achieving histologic-endoscopic mucosal improvement during the induction phase.

**Supplementary Figure 5. Forest plot of symptomatic remission during induction phase.** Comparison of the effect of IL-23 inhibitors versus placebo on achieving symptomatic remission during the induction phase.

**Supplementary Figure 6. Forest plot of efficacy outcomes in the subgroup of patients with prior inadequate response or intolerance to biologics or JAK inhibitors.** Outcomes include (A) endoscopic improvement, (B) endoscopic normalization, (C) symptomatic remission, and (D) histo-endoscopic mucosal improvement with IL-23 inhibitors versus placebo during induction therapy.

**Supplementary Figure 7. Forest plot of serious infection.** Comparison of the incidence of serious infection between patients treated with IL-23 inhibitors and placebo during the induction phase.

**Supplementary Figure 1**


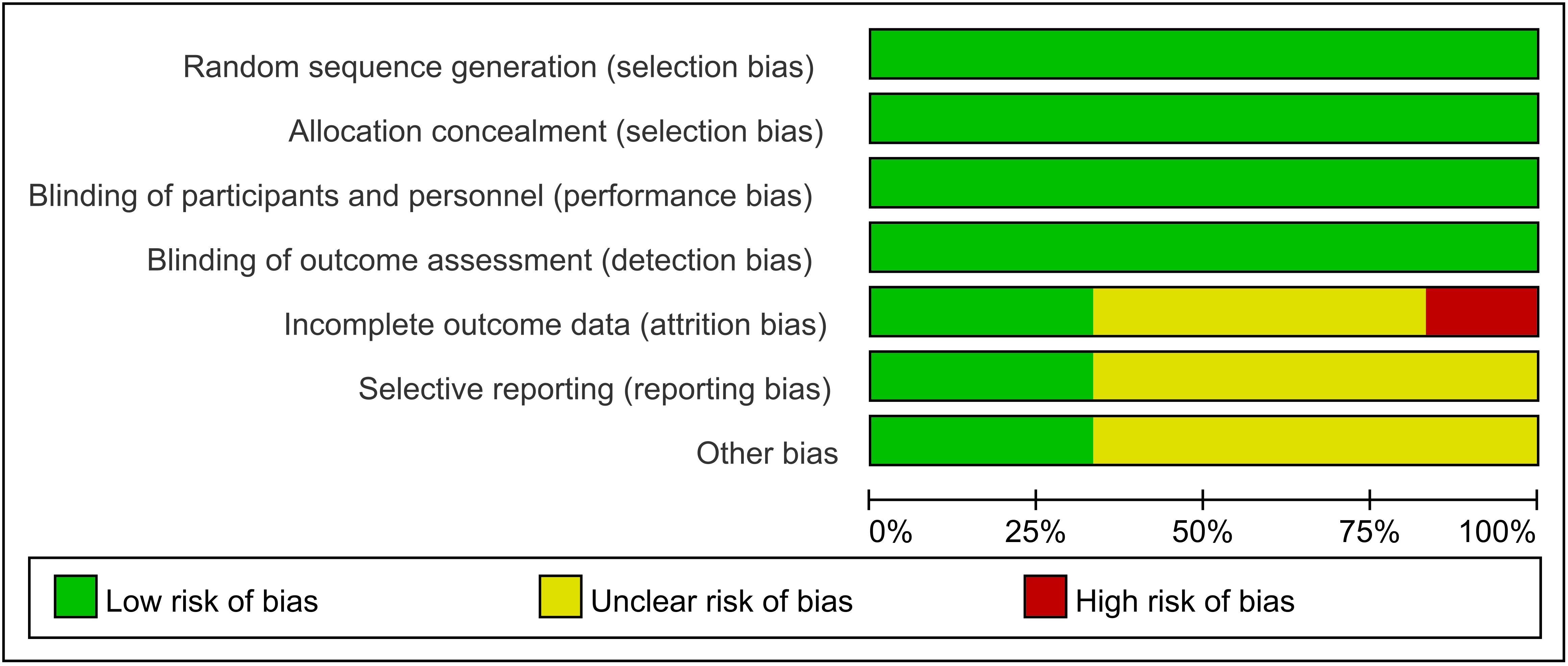


**Supplementary Figure 2**


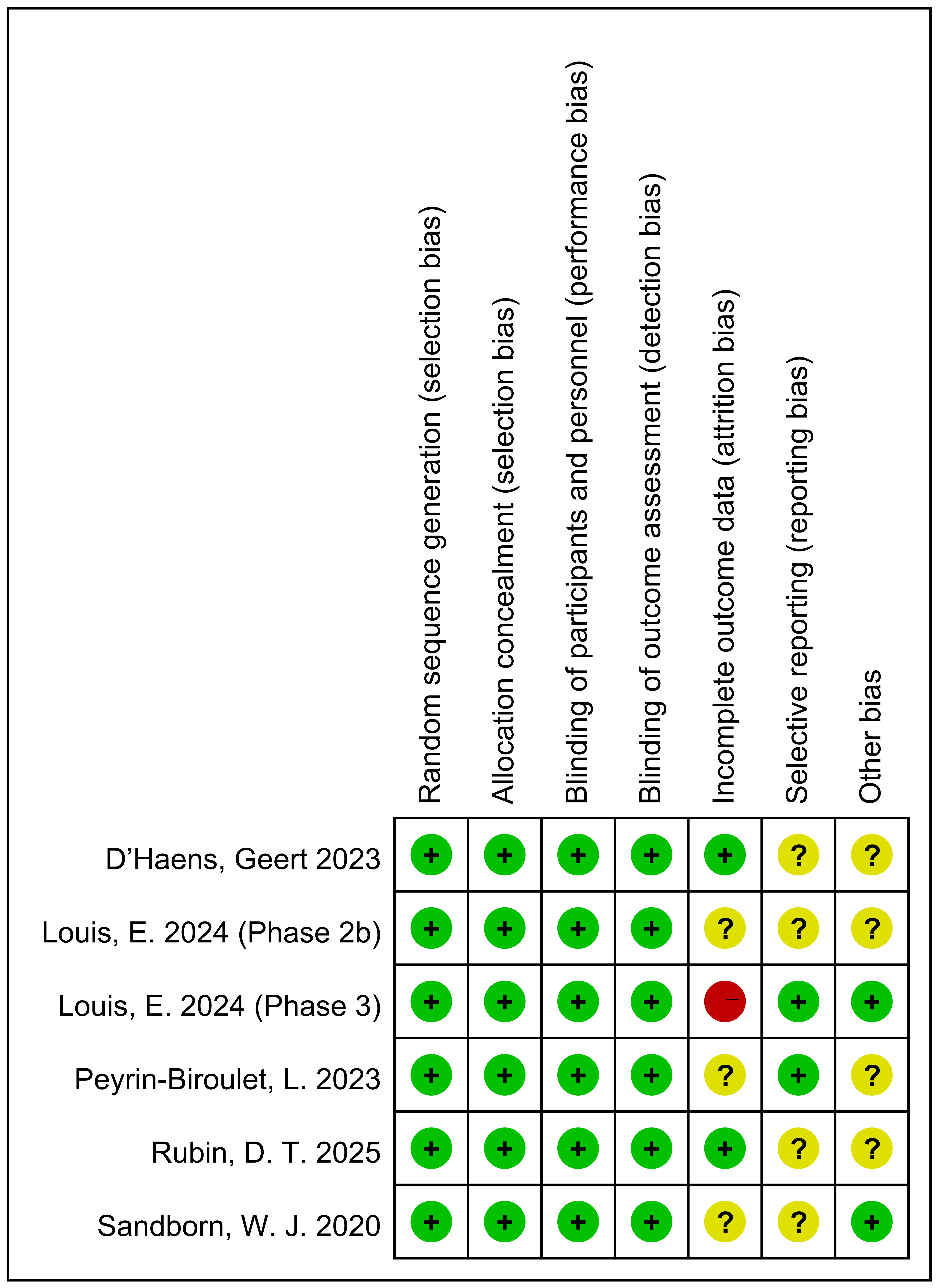


**Supplementary Figure 3
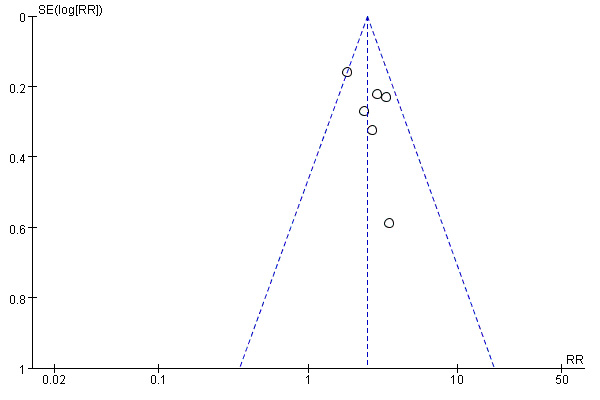
**

**Supplementary Figure 4**


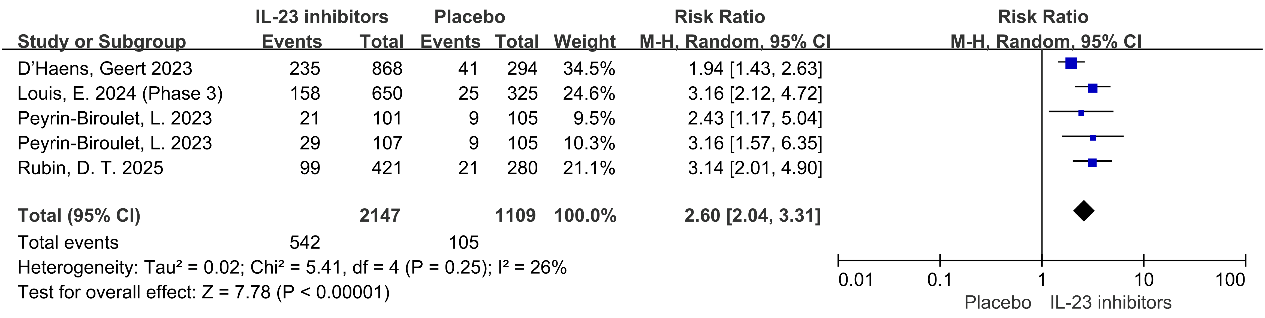


**Supplementary Figure 5**


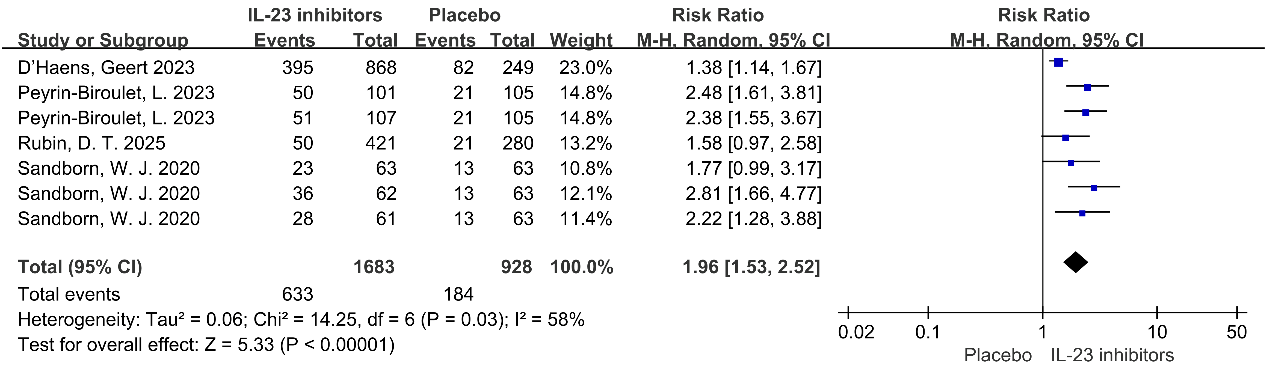


**Supplementary Figure 6**


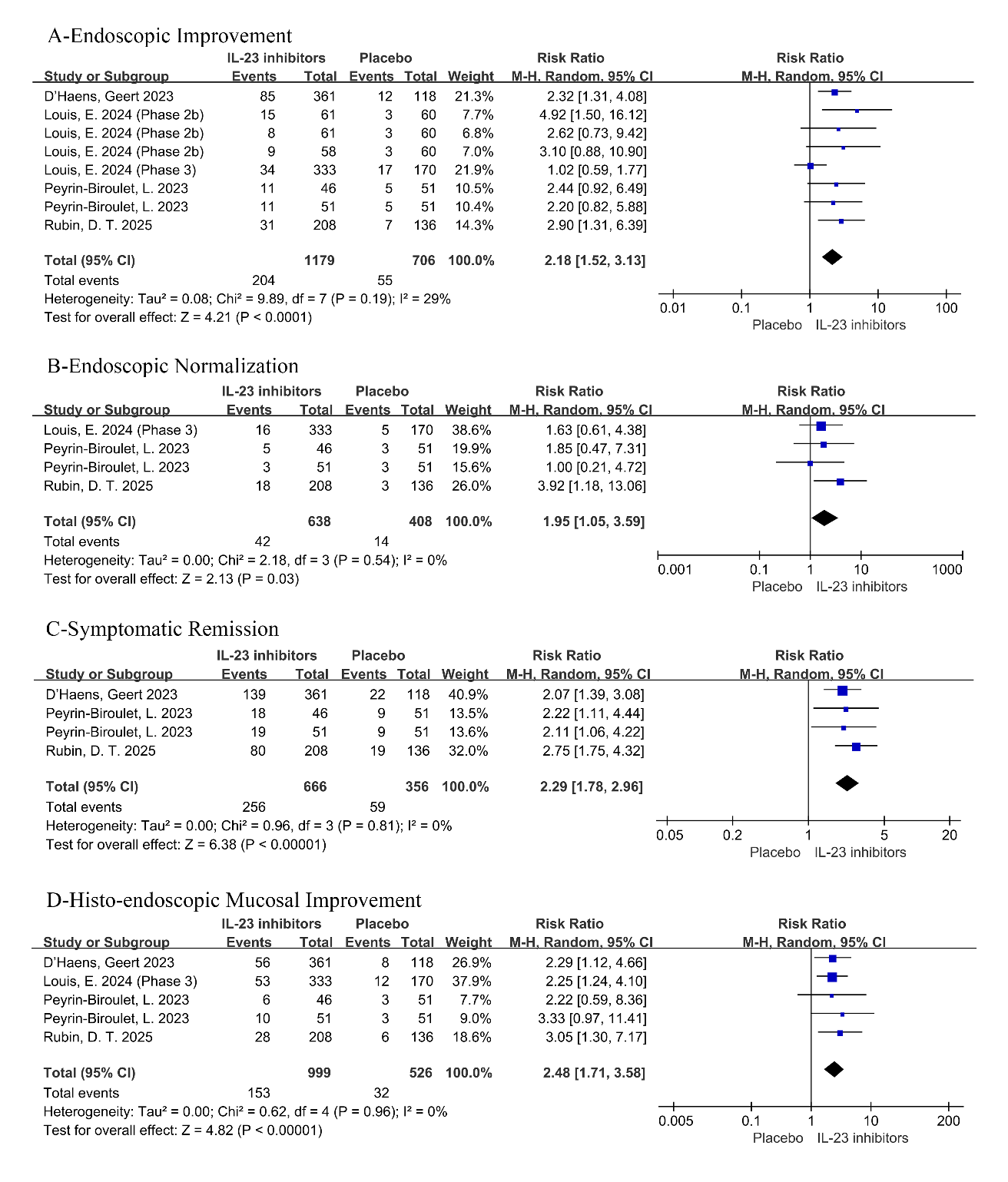


**Supplementary Figure 7**


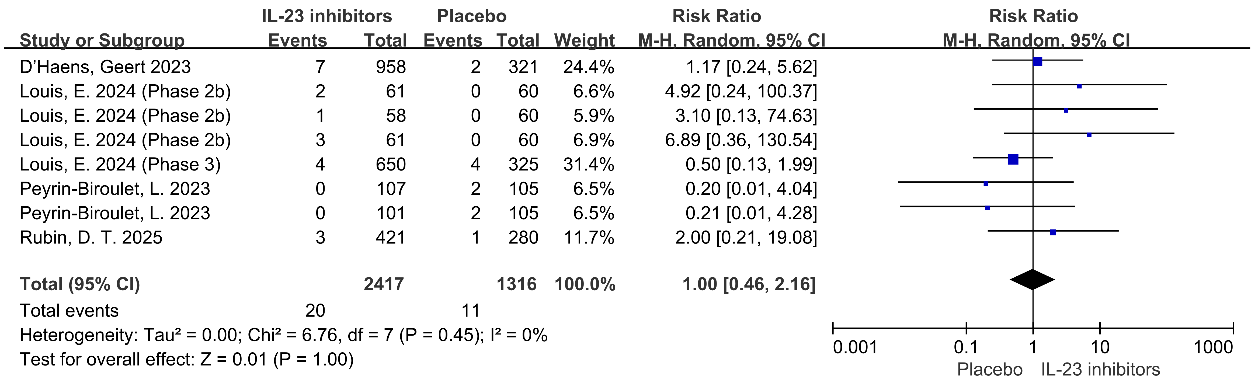

Supplement: Supplementary file 1 [file Supplementary_Information.docx]
